# Assessing the HIV-1 Epidemic in Brazilian Drug Users: A Molecular Epidemiology Approach

**DOI:** 10.1371/journal.pone.0141372

**Published:** 2015-11-04

**Authors:** Monick Lindenmeyer Guimarães, Bianca Cristina Leires Marques, Neilane Bertoni, Sylvia Lopes Maia Teixeira, Mariza Gonçalves Morgado, Francisco Inácio Bastos, Brazilian Multicity Study Group on Drug Misuse

**Affiliations:** 1 Laboratório de AIDS e Imunologia Molecular, Instituto Oswaldo Cruz- FIOCRUZ, Rio de Janeiro, Brasil; 2 Instituto de Comunicação e Informação Científica e Tecnológica em Saúde- FIOCRUZ, Rio de Janeiro, Brasil; St. James School of Medicine, ANGUILLA

## Abstract

Person who inject illicit substances have an important role in HIV-1 blood and sexual transmission and together with person who uses heavy non-injecting drugs may have less than optimal adherence to anti-retroviral treatment and eventually could transmit resistant HIV variants. Unfortunately, molecular biology data on such key population remain fragmentary in most low and middle-income countries. The aim of the present study was to assess HIV infection rates, evaluate HIV-1 genetic diversity, drug resistance, and to identify HIV transmission clusters in heavy drug users (DUs). For this purpose, DUs were recruited in the context of a Respondent-Driven Sampling (RDS) study in different Brazilian cities during 2009. Overall, 2,812 individuals were tested for HIV, and 168 (6%) of them were positive, of which 19 (11.3%) were classified as recent seroconverters, corresponding to an estimated incidence rate of 1.58%/year (95% CI 0.92–2.43%). Neighbor joining phylogenetic trees from *env* and *pol* regions and bootscan analyses were employed to subtype the virus from132 HIV-1-infected individuals. HIV-1 subtype B was prevalent in most of the cities under analysis, followed by BF recombinants (9%-35%). HIV-1 subtype C was the most prevalent in Curitiba (46%) and Itajaí (86%) and was also detected in Brasília (9%) and Campo Grande (20%). Pure HIV-1F infections were detected in Rio de Janeiro (9%), Recife (6%), Salvador (6%) and Brasília (9%). Clusters of HIV transmission were assessed by Maximum likelihood analyses and were cross-compared with the RDS network structure. Drug resistance mutations were verified in 12.2% of DUs. Our findings reinforce the importance of the permanent HIV-1 surveillance in distinct Brazilian cities due to viral resistance and increasing subtype heterogeneity all over Brazil, with relevant implications in terms of treatment monitoring, prophylaxis and vaccine development.

## Introduction

Since the beginning of the AIDS epidemic in the early eighties, 757,042 AIDS cases have been reported in Brazil by the Brazilian Ministry of Health [1]. Brazil has an overall low general prevalence (0.4%), that seems to be stable since 2004 [2], but prevalence is substantially higher in key populations at higher risk. According to the UNAIDS criteria Brazilian epidemic is concentrated [3]. Diverse Brazilian surveys conducted from 1998 to 2009 in key subpopulations were assessed by a meta-analysis, with the results as follows: men who have sex with men [13.6 (95% CI: 8.2–20.2)], female sex workers [6.2 (95% CI: 4.4–8.3)] and heavy (illicit) drug users [23.1 (95% CI: 16.7–30.2)] [4].

In the first two decades of the AIDS epidemic in Brazil (1980–1997), people who injected drugs contributed with 17.3% of the AIDS cases, but since 1998, the prevalence of HIV infection in this population has been decreasing. A more pronounced decline was observed in recent years and nowadays corresponds to 2.1% of new AIDS cases [2]. This trend has yet to be fully elucidated, but seems to result from a combination of different factors such as spontaneous/secondary behavioral changes; the timely implementation of preventive measures (e.g. needles and syringes exchange programs) coupled with different programs aiming to reduce drug-related harm; the very low quality of street cocaine, making injection difficult and risky; and the marked transition to non-injectable routes, especially crack cocaine [5,6], as well as the pervasive violence of drug scenes and the associated high mortality that decimated the older cohorts of drug users (many of them injectors) over the years [7].

A national survey conducted in 2005 revealed a very low (<0.1%) prevalence of active injecting drug users in a representative sample of the Brazilian urban population. On the other hand, the same survey documented high consumption rates of licit (alcohol) and illicit drugs (especially marijuana, cocaine and solvents) [8].

Active drug users usually have lower adherence to HIV-1 treatment (60%) than former (68%) and non-users (77%) [9]. Structural (social marginalization, financial constraints, incarceration) and individual (anxiety and depression, other comorbidities) barriers to optimal adherence were reviewed by Wood et al. [10]. These factors, together with their uneven access to health services and risky sexual behavior (unprotected sex, sexual partner turnover and engagement in sex work/sex vs. drug exchange) [11], suggest drug users may have a key role in the overall dynamics of HIV dissemination, including viral genomes bearing drug resistance mutations (DRMs) [12]. However, some recent studies did not make evident any differential of people who inject drugs living with HIV to develop antiretroviral resistance in relation to other HIV-positive populations [13,14].

Three molecular epidemiology studies were conducted in Brazil among people who injected drugs in the 1990s, two in Rio de Janeiro (1994-1997/1999-2001) [15,16] and one in São Paulo [17]. In Rio de Janeiro, a decrease from 26.3% to 7.9% of HIV point prevalence was observed over time, associated with an increase in the incidence from 0% to 0.76% [15,16] and a high prevalence of drug resistance mutations [18]. Moreover, a higher prevalence of sub-subtype F1 and BF1 recombinants was observed in this population, compared to other key populations at higher risk from both cities [17,18].

In spite of the transition of the preferential self-administration route from injectable to non-injectable drugs in place since the 1990s, little is known about the characteristics of this population of heavy drug users under fast transition, in part due to the persistent marginalization of this group that tends to be a “hidden population”, which is hard to recruit and estimate. Classic probability sampling methods are not appropriate to recruit and estimate this evasive and scattered group of people. In order to overcome these methodological and logistic caveats, many different methods have been proposed to better estimate this and other hidden populations, such as Respondent-driven sampling (RDS).

Thus, the present study aimed to assess HIV-1 infection rates (estimated incidence and prevalence), as well as the molecular epidemiology, the frequency of DRM, and the identification of HIV transmission clusters among heavy drug users recruited in eight different Brazilian cities, in a RDS-based study, carried out in 2009.

## Materials and Methods

### Study population

Our study population was composed by individuals aged 18 or older, defined as “people who use heavy drug” (DUs) by the “CODAR Criteria” of Pan-American Health Organization as individuals who “had injected drugs at least once in the last six months” and/or “had used cocaine, crack, opiates, or illicit drugs [other than marijuana/hashish] by other routes [snorted, smoked, ingested] for at least 25 days in the last 6 months” [19].

To recruit participants, we used RDS. RDS is a chain-referral sampling method for the recruitment of hard-to-reach individuals with the help of successive nominations by individuals belonging to the same population, who comprised both the seeds (initial recruiters), as well as the members of the successive recruited waves.

The study, funded by the Brazilian Ministry of Health, took place in 10 Brazilian cities, in 2009, and 3,487 heavy drug users were recruited. The participants answered a self-administrated questionnaire using ACASI (Audio Computer-Assisted Self Interviewing)) and HIV and syphilis rapid tests were performed (see “HIV serology” below). Dried spots with biological material were obtained for these individuals after HIV testing, HIV-1 seropositive individuals were invited to donate plasma and whole blood samples. The biological samples from the site of Rio de Janeiro were received and processed in the Laboratory of AIDS and Molecular Immunology (LABAIDS), at the Oswaldo Cruz Institute (IOC/FIOCRUZ), Rio de Janeiro, Brazil, in parallel with the field work, whereas the other sites maintained biological materials (whole blood, plasma and dried spots) stored until the end of the study. In that occasion, this material was transported in dry ice to LABAIDS according to international biosafety rules, for molecular analyses.

This material was analyzed and eight of these 10 selected cities provided valid biological samples [Rio de Janeiro (Rio de Janeiro/RJ); Belo Horizonte (Minas Gerais/MG); Recife (Pernambuco/PE); Salvador (Bahia/BA); Brasilia (Distrito Federal/DF); Campo Grande (Mato Grosso do Sul/MS); Itajaí (Santa Catarina/SC) and Curitiba (Paraná/PR) and took part of the present study. In these eight cities, 2,878 individuals were eligible. However, some individuals did not agree to be tested for HIV-1. Excluding these individuals, as well as those with an indeterminate HIV-1 serology, the overall findings presented here refer to 2,812 individuals.

### Ethics

This study was approved by the Oswaldo Cruz Foundation Ethical Research Committee (0114.031.000–0 and 0044.0.031.000–09) and written consents was obtained from all studied individuals.

### Sociodemographic and behavioral data analysis

For the purpose of the present paper, sociodemographic and behavioral variables were cross-tabulated using contingency tables and descriptive statistics, without taking in consideration the underlying network structure.

Network data were analyzed using Stata v.13 and NetDraw 2.091. The latter was used to generate network diagrams of interviewees recruited in each site, and depict their basic characteristics.

### HIV serology

HIV serology was performed using two rapid tests; Rapid Check HIV 1 & 2 (NDI—Núcleo de Doenças Infecciosas, Vitória, ES, Brasil) and DPP^®^ HIV-1/2 Bio-Manguinhos (Fundação Oswaldo Cruz/Bio-Manguinhos, Rio de Janeiro, Brasil), according to the manufacturer’s instructions.

### HIV-1 incidence determination

In order to estimate HIV-1 recent infections, plasma samples were subjected to a quantitative competitive capture enzyme immunoassay—Calypte HIV-1 BED Incidence EIA (Calypte Biomedical Corporation, USA) [20]. Briefly, in this assay, once plasma is added, anti-HIV-1 IgG and non-anti-HIV-1 IgG populations are captured on goat-anti-human IgG coated wells, and the relative amount of anti-HIV-1 IgG captured is measured. Samples with an optical density (OD) <0.8 of calibrator OD are classified as recent infections (<6 months since infection). Annual HIV seroincidence and 95% confidence intervals (CIs) were calculated using the 155-day window period and the standard seroincidence formula as described elsewhere [20].

### Amplification and sequencing of HIV-1 DNA

DNA samples were extracted from 200 ul of whole blood using the QIAamp DNA kit (Qiagen Inc., CA, U.S.A.), according to the manufacturer’s protocol. Amplification of the *PR/RT* and *C2-V3env* region was performed as described elsewhere [21,22]. The PCR products were purified using the Illustra GFX PCR DNA purification kit (GE Healthcare) according to the manufacturer’s protocol. Purified DNA was sequenced using the ABI Big Dye Terminator v.3.1 cycle sequencing ready reaction kit (Applied Biosystems, CA, U.S.A.), and processed with an automated ABI 3100 Genetic Analyzer (Applied Biosystems).

### Subtype classification

Nucleotide sequences were aligned using the Clustal X program [23] implemented in Mega 5.2 program and later manually edited. All positions with alignment gaps were removed, resulting in two alignments, one corresponding to the *PR/RT* regions of *pol* gene (nucleotides 2265–3519 relative to HXB2); and the second ones corresponding to the *C2-V3 env* region (nucleotides 6922–7277 relative to HXB2). References sequences from all CRF_BF circulating in South America which present recombination breakpoint in the regions under study were included in the analysis. The Maximum Likelihood (ML) phylogenetic trees *pol* and *env* were inferred under the GTR+I+Γ4 nucleotide substitution model, selected using the jModeltest program [24]. The ML tree was reconstructed with the PhyML program [25] using an online web server [26]. Heuristic tree search was performed using the SPR branch-swapping algorithm and the reliability of the obtained topology was estimated with the approximate likelihood-ratio test (aLRT) [27] based on the Shimodaira-Hasegawa-like procedure. The ML trees were visualized using the FigTree v1.3.1 program available at: http://treebioedacuk/software/figtree.

Recombination analysis was performed by bootscan analysis as implemented in the Simplot version 3.5.1 [28], using representative sequences of HIV-1 subtypes B, C, and F1 as reference. Bootstrap values supporting branching with reference sequences were determined in Neighbor-Joining trees constructed using the K2-parameter model [29], based on 100 resamplings, with a 200nt sliding window moving in steps of 10 bases.

### Transmission networks analysis

In order to evidence the putative transmission network of those RDS clusters detected in the subtyping classification (ML *env* and *pol*), new ML analyses were performed. For these analyses we used the HIV basic local alignment search tool (HIV BLAST) (http://www.hiv.lanl.gov/content/sequence/BASIC_BLAST/basic_blast.html) to select *env* and *pol* reference sequences with the highest similarity score (>90%) to each group of RDS samples that clustered together in the subtyping trees. Additionally, Brazilian sequences with assign risk group belonging to subtypes B and C were also included in both studied regions. In total we aggregate to the sequences herein studied (n = 18) some Brazilian subtypes B, C and F1 sequences (n = 222 *pol* / 249 *env*) and non-Brazilian reference sequences (n = 12 *pol* or *envpol*). The same parameters used in the ML described above were used in the reconstruction of these trees.

### Drug resistance mutations analysis

All *PR/RT* sequences were evaluated for transmitted drug resistance mutations (TDRM). This analysis was performed according to the Calibrated Population Resistance Tool (CPR Version 6.0) that uses the Surveillance Drug Resistance Mutation panel 2009 of the Stanford genotypic resistance interpretation algorithm [30].

### Data Availability

All nucleotide sequences are available from the GenBank database (accession numbers *POL*- KT355891—KT356022, *ENV*-KT356023—KT356154.

## Results

In the eight cities under analysis, most interviewees who agreed to be rapid tested for HIV (78.5%) were male and had less than 34 years old (70.7%). The use of injected drugs was reported by 12.5% of the individuals, and more than half of participants (53.5%) informed never had been previously tested for HIV. Among this, 2,812 individuals, 168 (6.0%) had their seropositive status confirmed by subsequent tests, and the HIV prevalence was higher among those with a history of currently injecting drugs (9.9%) than among non-injecting drug users (5.4%) (Table 1). The highest HIV seroprevalence was 10.0%, made evident in Itajaí (SC) and the lowest ones were found in the two Northeastern cities, Recife (PE) and Salvador (BA), with 4.7% and 4.3%, respectively (Table 1).

**Table 1:** Socio-demographic and clinical characteristics of drug users recruited by RDS method and screened to HIV in eight Brazilian cities, 2009.

Brazilian Cities									
Variables	Recife	Salvador	Brasilia	Campo Grande	Belo Horizonte	Rio de Janeiro	Curitiba	Itajaí	Overall
	(n = 361)	(n = 422)	(n = 280)	(n = 199)	(n = 348)	(n = 606)	(n = 297)	(n = 299)	(n = 2812)
Sex (%)									
Male	273	289	192	182	298	443	257	248	2182
	(75.6)	(72.1)	(69.1)	(92.4)	(86.9)	(73.6)	(86.5)	(82.9)	(78.5)
Female	88	112	86	15	45	159	40	51	596
	(24.4)	(27.9)	(30.9)	(7.6)	(13.1)	(26.4)	(13.5)	(17.1)	(21.5)
Age (years) (%)									
18–24	145	162	93	56	69	163	94	86	868
	(40.6)	(38.4)	(33.2)	(28.4)	(19.9)	(26.9)	(31.6)	(28.9)	(31.0)
25–34	135	170	109	72	158	210	134	125	1113
	(37.8)	(40.3)	(38.9)	(36.6)	(45.5)	(34.7)	(45.1)	(41.9)	(39.7)
35–49	68	80	71	63	107	169	63	64	685
	(19.0)	(19.0)	(25.4)	(32.0)	(30.8)	(27.9)	(21.2)	(21.5)	(24.4)
≥ 50	9	10	7	6	13	63	6	23	137
	(2.5)	(2.4)	(2.5)	(3.0)	(3.7)	(10.4)	(2.0)	(7.7)	(4.9)
Drug injection (%)									
Yes	12	24	31	13	84	112	35	33	344
	(3.3)	(6.0)	(11.2)	(6.7)	(24.6)	(18.7)	(11.9)	(11.1)	(12.5)
No	347	375	237	174	246	465	247	260	2351
	(96.4)	(93.5)	(85.9)	(90.2)	(71.9)	(77.6)	(84.0)	(87.5)	(85.1)
Non declared	1	2	8	6	12	22	12	4	67
	(0.3)	(0.5)	(2.9)	(3.1)	(3.5)	(3.7)	(4.1)	(1.3)	(2.4)
HIV-1 positive Serology (%)	17	18	14	10	21	40	18	30	168
	(4.7)	(4.3)	(5.0)	(5.0)	(6.0)	(6.6)	(6.1)	(10.0)	(6.0)
Prevalence of HIV according to type of the drug use (%)									
NIDU	17/347	14/375	13/237	7/174	12/246	25/465	13/247	25/260	126/2351
	(4.9)	(3.7)	(5.5)	(4.0)	(4.9)	(5.4)	(5.3)	(9.6)	(5.4)
IDU	0/12	2/24	1/31	2/13	8/84	12/112	4/35	5/33	34/344
	(0.0)	(8.3)	(3.2)	(15.4)	(9.5)	(10.7)	(11.4)	(15.2)	(9.9)
Previous HIV testing (%)									
Yes	117	154	151	76	168	267	171	182	1286
	(32.4)	(38.4)	(54.5)	(39.2)	(49.0)	(44.6)	(58.0)	(61.1)	(46.5)
No	244	247	126	118	175	332	124	116	1482
	(67.6)	(61.6)	(45.5)	(60.8)	(51.0)	(55.4)	(42.0)	(38.9)	(53.5)
HIV incidence (%)**	1.36	1.73	0.88	2.45	2.84	2.06	0	1.74	1.68
Incident	2	3	1	2	4	5	0	2	19
	(11.8)	(16.7)	(7.1)	(20.0)	(19.0)	(12.5)	(0.0)	(6.7)	(11.3)
Prevalent	15	15	13	8	17	35	18	28	149
	(88.2)	(83.3)	(92.9)	(80.0)	(81.0)	(87.5)	(100)	(93.3)	(88.7)

** HIV-1 incidence is expressed in % per year.

IDU-injecting drug users, NIDU-non-injecting drug users. The numbers in the table did not necessarily add up to the total number of samples due to missing information.

Nineteen among the 168 seropositive individuals (11.3%) were classified as recent seroconverters, corresponding to an estimated incidence rate of 1.68%/year (95% CI 0.92–2.43%). The highest HIV-1 incidence rates were found in Campo Grande (MS) (2.45%/year) and Belo Horizonte (MG) (2.84%/year), whereas none incident cases were found in Curitiba (PR) (Table 1). The other five cities presented HIV-1 incidence rates ranging from 0.88%/year to 2.06%/year.

Approximately eighty percent (132 individuals) of the HIV-1 positive samples were subtyped in two genomic regions (*env/pol*). ML phylogenetic *pol*, *env* and recombination analyses (S1–S3 Figs) were able to classify 66 (50.0%) of the samples as subtype B, 28 (21.2%) as BF recombinants, 27 (20.4%) as subtype C, 5 (3.8%) as subtype F1, 2 (1.5%) as BC recombinants, 1 (0.8%) as subtype D and three samples (2.3%) presented an unclassified fragment (UF1*pol* /F1env, CBU*pol/* Cenv, FBU*pol/* B*env*) (S3 Fig). From those 28 BF recombinants three samples could be classified as CRF28/CRF29_BF-like (RDSBA198, RDSBA393 and RDSRJ609), since they present the same pattern of recombination of this CRF (BF*pol*/B*env*). The samples RDSSC86, RDSDF206 and RDSMS226 were a second generation of CRF28/29, as they had an additional recombination breakpoint in *pol* (S3 Fig).

**Fig 1:**
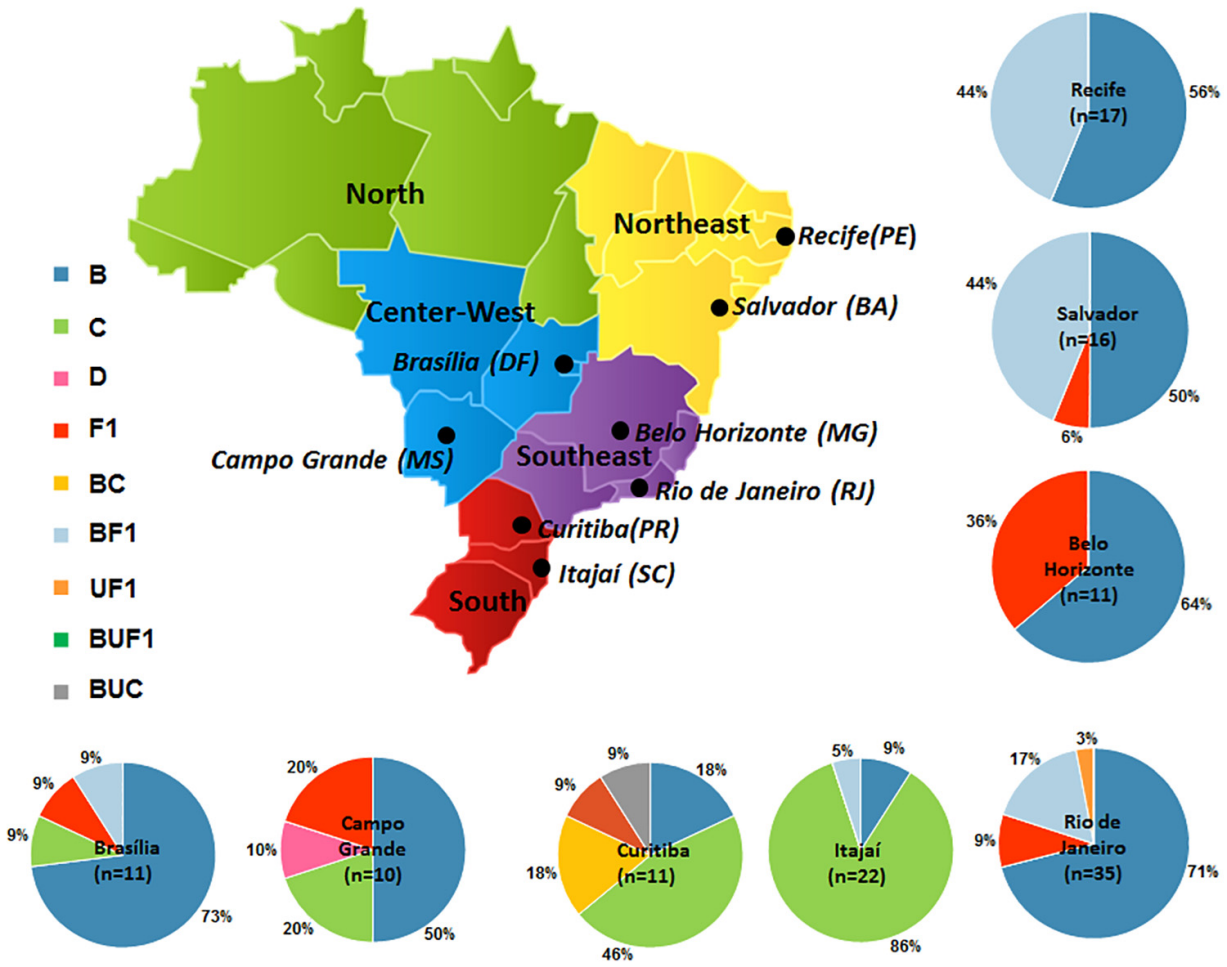
Map of Brazil showing the frequency of HIV-1 genetic variants across the nine studied cities/State [Recife (PE), Salvador (BA)—Northeast region], [Rio de Janeiro (RJ), Belo Horizonte (MG)—Southeast region], [Itajaí (SC), Curitiba (PR)—South Region], [Campo Grande (MS), Brasilia (DF)—Center West region]. The number of *env* and *pol* analyzed samples was depicted in each graphic.

The distribution of subtypes according to the different Brazilian cities is depicted in Fig 1. Subtype C strains were prevalent in both Southern cities (Itajaí and Curitiba). Outside this region it was also detected in Campo Grande (20.0%) and Brasília (9.0%) (Center-West region), whereas BC recombinants (18.0%) were exclusively found in Curitiba (South region). BF recombinants were detected in all studied cities with the exception of Itajaí and subtype D was documented in Campo Grande. The typical Brazilian subtype B (GWG at the top of V3 loop) was detected in half of the cities, corresponding to 12.5%, 33.0%, 35.0% and 50.0% of subtype B infections in Brasília, Belo Horizonte, Rio de Janeiro and Curitiba, respectively (data not shown).

**Fig 2:**
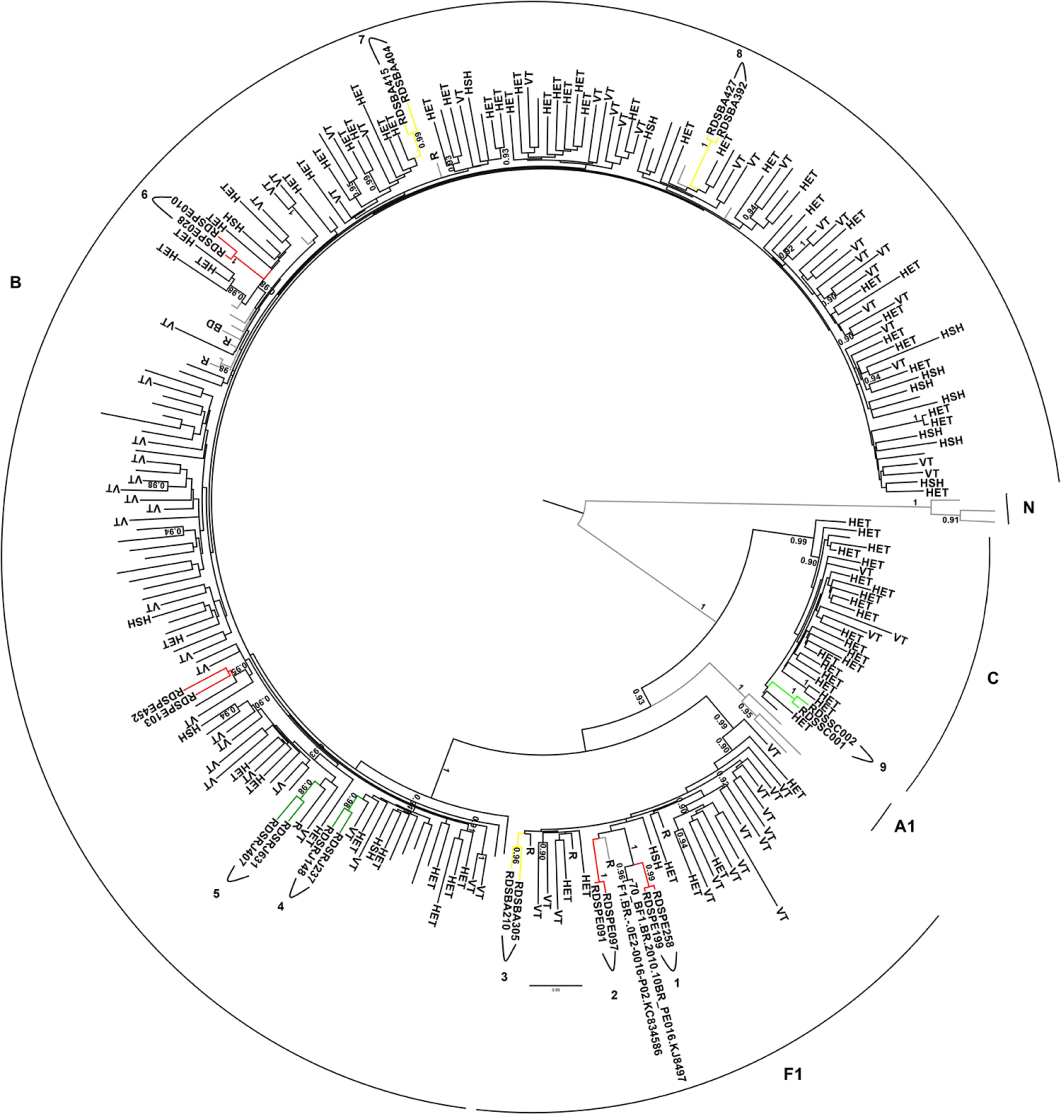
*Pol* ML tree (2265–3519 relative to HXB2) from Brazilian RDS drug users clusters and reference sequences with highest similarity score to each group selected by HIV BLAST, as well as, Brazilian sequences with assigned transmission category [heterosexual (HET), homosexual (HSH), blood donor (BD) or vertical transmission (VT)] belonging to subtypes B, C and F1. The *aLRT* support values superior to 0.90 are indicated. Horizontal branch lengths are drawn to scale with the bar at the bottom indicating nucleotide substitutions per site.

**Fig 3:**
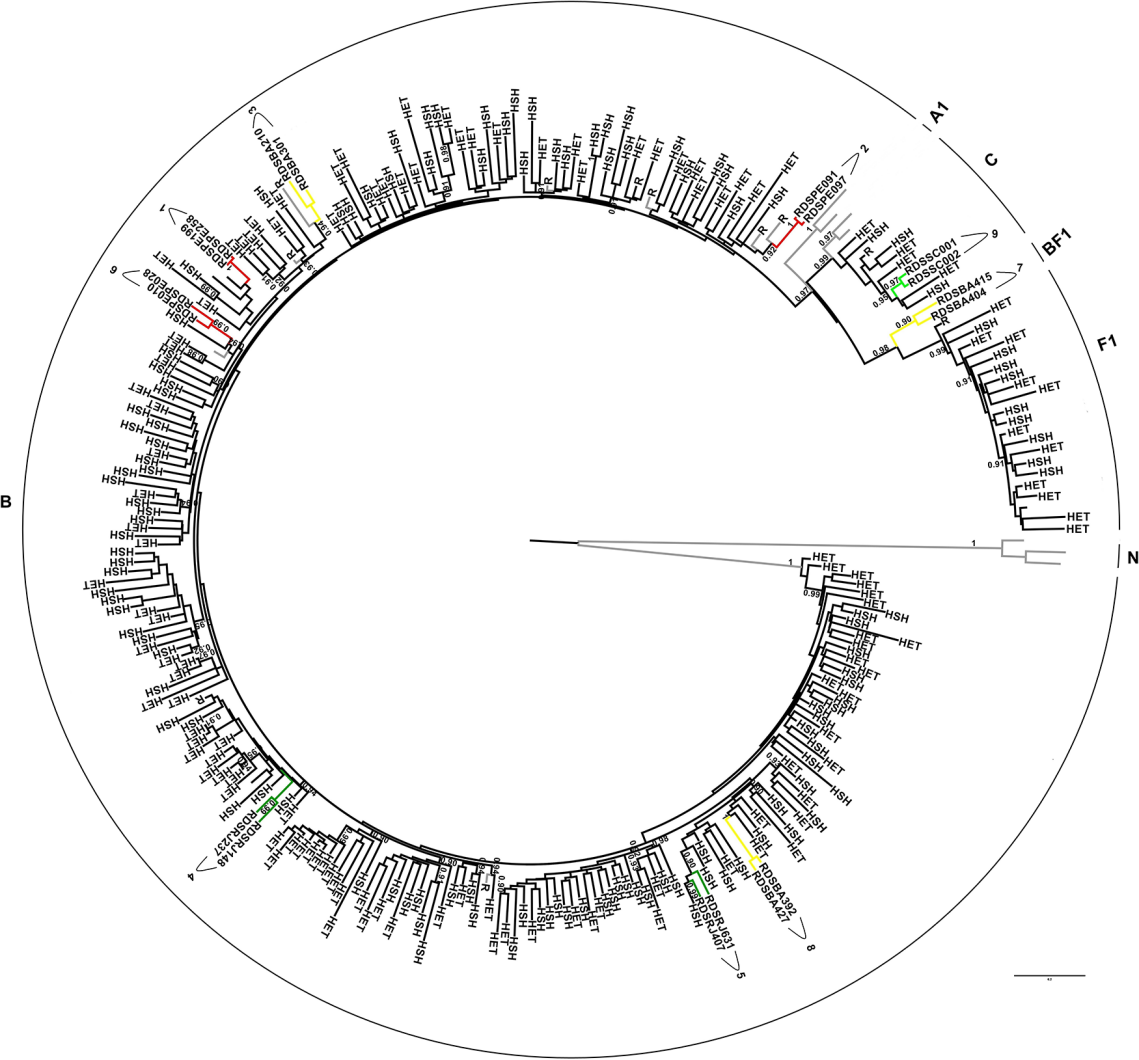
*Env* ML tree (6922–7277 relative to HXB2) from Brazilian RDS drug users clusters and reference sequences with highest similarity score to each group selected by HIV BLAST, as well as, Brazilian sequences with assigned transmission category [heterosexual (HET), homosexual (HSH), blood donor (BD) or vertical transmission (VT)] belonging to subtypes B, C and F1. The *aLRT* support values superior to 0.90 are indicated. Horizontal branch lengths are drawn to scale with the bar at the bottom indicating nucleotide substitutions per site.

Based on ML phylogenetic *pol* and *env* (S1 and S2 Figs) nine groups composed by two sequences with aLRT ≥0.90 were detected. In order to testify the relationship among these nine DU clusters, additional Brazilian and non-Brazilian reference sequences selected based on the BLAST similarity, as well as Brazilian sequences with assigned transmission categories (heterosexual, homosexual, blood donor or vertical transmission) belonging to subtypes B, C and F1 were included in new ML analyses (Fig 2–*pol* and Fig 3-*env*), (see material and methods section). These phylogenetic analyses confirmed the relationship among these nine groups. Further, some sequences obtained through BLAST searches cluster with sequences from the current study. In *pol* ML tree the cluster 1 branched together with two Brazilian reference samples also from PE with aLRT of 1. The same observation could be made for other studied clusters in the *env* ML; cluster 5 that branched together with two other samples from RJ, and clusters 2 and 3 that grouped with non-Brazilian samples, all of them with aLRT scores ≥0.90.

**Fig 4:**
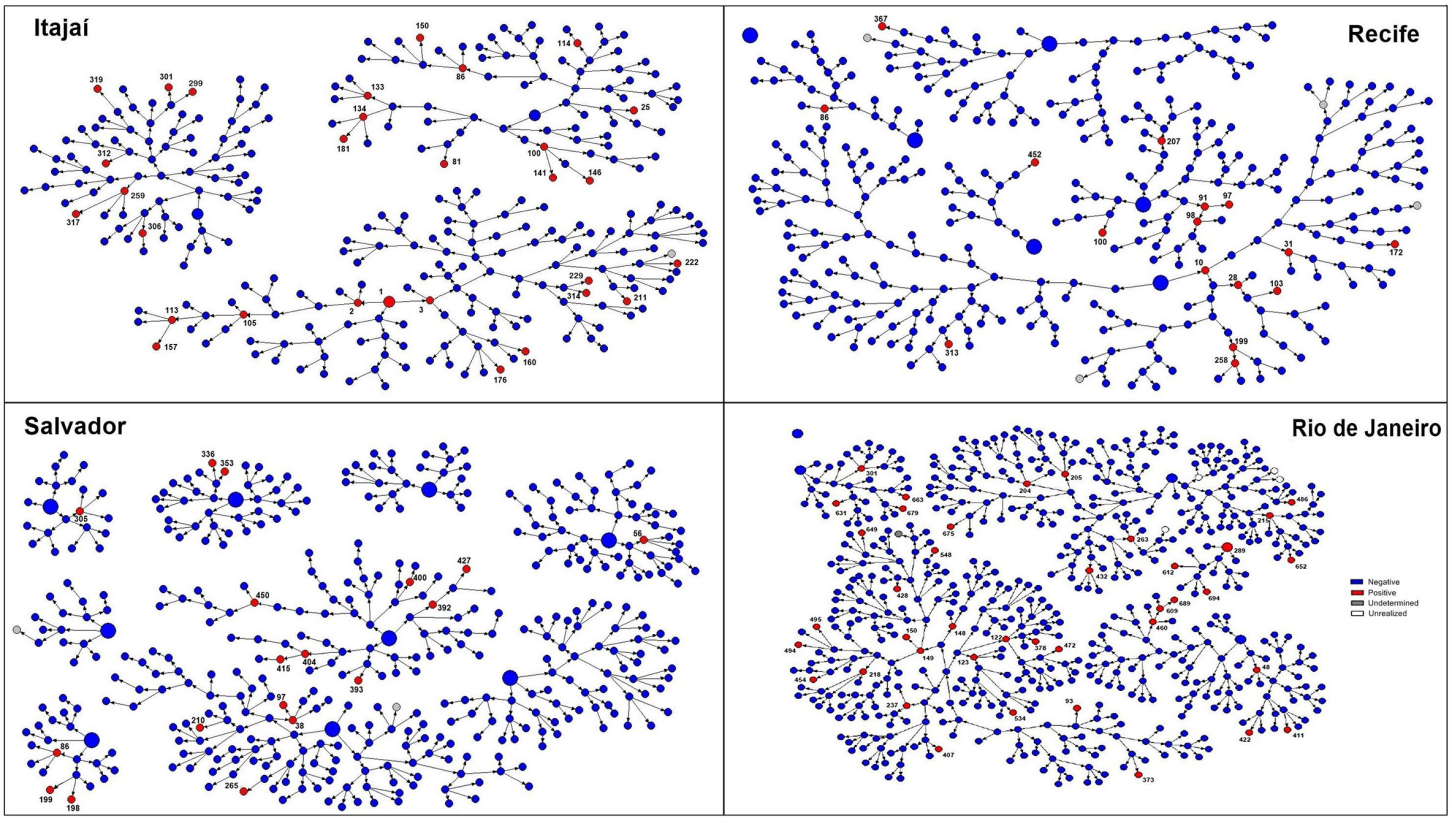
Schematic representation of responded driven sampling (RDS) recruitment network from Itajaí (SC), Recife (PE), Salvador (BA) e Rio de Janeiro (RJ), places were the phylogenetic cluster were detected. Large circles represent the first recruited individual in each network, designated as seeds and small circles represent the consecutive waves of recruitment. HIV-1 positive individuals were colored in red, HIV-1 negative individuals in blue and individuals not tested for HIV in gray. The numbers were depicted only in HIV-1 positive individuals and were related to the sequence numbers (e.g. individual marked as number 1 in Itajai RDS representation corresponds to the RDSSC001 sequence).

Some of the clusters detected in phylogenetic analyses were coincident with the networks made evident by the RDS chain-referral structure, as exemplified in Fig 4, where all three groups of Recife (PE) samples detected (1,2, 6) were directly connected in the recruitment network. On the other hand, among the two groups of individuals from Rio de Janeiro (RJ) discriminated by the phylogenetic analyses, one of them belong to the same RDS network recruitment, although not directly connected (4), whereas a second subgroup of samples (5) belonged to a distinct recruitment network. Three groups of Salvador (BA) sequences were detected (3, 7, 8). In two of them (7 and 8) the individuals were recruited by the same network component, and direct connection was only made evident in the group 7. The BA group (3) was composed by individuals recruited in distinct networks. The group nine composed by two individuals from Itajaí (SC) was directly connected through the RDS recruitment network (Fig 3).

One hundred and thirty-two individuals were assessed for PR/RT drug resistance analysis; however, hypermutation was detected in three PR and one RT sequences, resulting in their exclusion from the final analysis. Drug resistance mutations were found in 16 (12.2%) of the samples under analysis. Mutations related to protease inhibitors were detected in 3.1% of the individuals and the most prevalent of them was L90M (2.3%). Resistance mutations related to NRTI and/or NNRTI were found in 6.1% of the samples, and the most prevalent mutations were M184V (2.3%) and K103N (3.8%), respectively to NRTI and NNRTI. The percentage of drug resistance mutations according to the studied city is very variable (Table 2). Two individuals from Rio de Janeiro (RDSRJ472 and RDSRJ150) presented complex NRTI and PI resistance mutation profiles, suggesting previous exposition to antiretroviral treatment. A complete list and percentage of drug resistance mutations detected, stratified by drug class is presented in Table 2.

**Table 2:** List and frequency of drug resistance mutations detected in each studied city stratified by drug class.

Drug resistance mutations (DRM) (%)					
Studied City	Sequences with DRM	Sequence	NRTI	NNRTI	PI
Salvador (n = 16)	1	RDSBA056	K219N	None	None
			**(6.3)**	**(0.0)**	**(0.0)**
Brasília (n = 11)	2	RDSDF112	None	K103N	None
		RDSDF290	None	K103N	None
			**(0.0)**	**(18.8)**	**(0.0)**
Campo Grande (n = 10)	2	RDSMS106	None	None	L90M
		RDSMS109	K219Q	K103N, G73S	L90M
			**(10.0)**	**(20.0)**	**(20.0)**
Recife (n = 16)	1	RDSPE091	K70R	None	None
			**(6.2)**	**(0.0)**	**(0.0)**
Curitiba (n = 11)	1	RDSPR089	None	K103N	None
			**(0.0)**	**(9.0)**	**(0.0)**
Rio de Janeiro (n = 35)	6	RDSRJ150	M41L, L210W, T215D	None	M46I, L90M
		RDSRJ237	None	Y188C	None
		RDSRJ373	M184V	K103N	None
		RDSRJ472	M41L, D67N, L210W, T215SY	None	None
		RDSRJ495	M184V	None	None
		RDSRJ612	None	G190A	None
			**(25.7)**	**(8.6)**	**(5.7)**
Itajaí (n = 22)	3	RDSSC105	None	None	M46I
		RDSSC114	M184V	None	None
		RDSSC211	None	V106M	None
			**(4.5)**	**(4.5)**	**(4.5)**
Belo Horizonte (n = 11)	0		**(0.0)**	**(0.0)**	**(0.0)**

DRM: Drug resistance mutations; NRTI: Nucleoside/Nucleotide Reverse Transcriptase Inhibitors; NNRTI: Non-nucleoside reverse-transcriptase inhibitors; PI: Protease inhibitors

## Discussion

In this study, moderately HIV-1 prevalence rates (6.0%) were detected in drug users from eight Brazilian cities, ranging from 4.3% in Salvador to 10.0% in Itajaí. These rates are 10 to 25 times higher than the estimated prevalence for the Brazilian general population 0.4% [2]. However, even considering the prevalence of 9.9% detected herein in the subgroup of people who injected drugs, such prevalence is significantly lower than what had been previously reported for this group in the beginning of the AIDS epidemic (early 1980s) 29.5% (95% CI: 20.0–39.9) [4], when this group represented a relevant fraction of the HIV-infected population.

Two studies targeting people who injected drugs in Rio de Janeiro in the 1990s found HIV-1 prevalence rates of 26.3% and 15.9% [15,31]. In the same period, higher rates of HIV-1 prevalence (65%) were also detected in a study conducted in Porto Alegre, Southern Brazil [31] and in two multicity studies in this key population at higher risk. The first one, AjUDE-Brasil I, conducted in 1998, in five Brazilian cities (São Paulo, Sorocaba, and São José do Rio Preto from the State of São Paulo; Itajaí (SC); and Porto Alegre, Rio Grande do Sul [RS]), and the second one, AjUDE-Brasil II, carried out in 2000–2001 in six cities (Salvador (BA); São José do Rio Preto (SP); Florianópolis and Itajaí (SC); Porto Alegre and Gravataí [RS]) found HIV-1 prevalences of 52.3% and 45.8%, respectively [32]. Later studies documented a decrease in the prevalence in this key population, as exemplified by a study conducted in 1999–2001 in Rio de Janeiro, where a prevalence of 11.7% in long-term and 4.3% in new injectors were found [33]. This relevant decrease seems to be associated to spontaneous behavior change as well as an integrated package of different interventions aiming to reduce drug-related harms. It is also important to point out to the shifting patterns of substance use, with a marked decline of the habit of injecting and the progressive increase of snorted and smoked varieties of cocaine. This dynamic was well documented in a study conducted among DU in São Paulo in three distinct periods (1991/92, 1994/96 and 1999). In this study, HIV-1 prevalence decreased from 63% to 42%, in tandem with a decline of the habit of injecting (from 42% to 15%) and the substantial increase of smoked crack cocaine, from 11% to 67% [34]. Although a substantial decline of HIV prevalence was observed when we compare the data from the present study with previous ones, moderately rates have been observed in both people who inject drugs (9.9%) and non-injecting drug users (5.4%). It is pivotal to maintain both preventative strategies aiming to curb HIV spread in this key population, as well as continuous surveillance [35].

The role of IDU in the HIV epidemic in neighbor Brazilian countries seems to be diverse. In the Andean countries (Venezuela, Colombia, Ecuador, Peru and Venezuela) the epidemic is concentrated in MSM and the role of IDU is negligible, contrasting with the dynamic observed in the Southern Cone (Paraguay, Argentina and Uruguay) [36]. In Argentina, the prevalence of HIV infection among this group ranged from 27% in 1987 to 63% in 1999 [37]. In 2002, accumulated AIDS cases in IDUs corresponds to 40%, even though, in the last notification of the Argentinean Ministry of Health concerning the period of 2011 to 2013, IDU corresponded to only 0.4% and 0.1% of the males and females cases, respectively [38]. In Paraguay, most AIDS cases are concentrated in Assunción and in the eastern frontier with Brazil and Argentina. In 2006 high HIV prevalence of 9.1% was detected in DU, contrasting with the prevalence of 0.3% among pregnant women. Similarly to Brazil and Argentina, the IDUs had an import role in the begging of the AIDS epidemic. It is important to note, that the relative number of female IDU is high in Uruguay, unlike the other Latin American countries [36].

Previous studies conducted by our group reported HIV-1 infection estimated incidences of 0% [15] and 0.76% [16] among people who injected drugs, and of 1.68%/year for people seeking HIV diagnosis at Voluntary and Counseling Centers of Rio de Janeiro [39], which are equal to that found in the present analysis (1.68%). On the other hand, an HIV-1 estimated incidence rate of 4.88% was found in a RDS based study targeting MSM from Campinas (SP) [40].

Herein, a predominance of HIV-1 subtype B (50–73%), followed by BF recombinants (9–44%) and subtype F (0–9%) was depicted in most geographic regions, with the exception of the southern cities, where HIV-1 subtype C (46–86%), BC or CBU recombinants (5–18%) and subtype B (9–18%) predominated. The presence of subtype C outside of the Southern cities was exclusively found in the Center-Western cities, Brasília (DF) and Campo Grande (MS). Its prevalence in Campo Grande was even higher than previously documented in a study including in drug naïve individuals (10.2%) [41]. Other studies have documented the progressive increment of subtype C in Goiânia (GO), another Center-Western city, where it accounted for 3.1% of the infections among pregnant women collected in 2003, whereas, in a study of 2013 with samples collected from 2008 to 2010, it corresponded to 11. 5% of the HIV-1 infections in the same study population [42,43]. Although the present study did not detect subtype C infections in the samples from the other Brazilian cities under analysis, other studies have found it HAART naïve individuals from São Paulo (11.6%) and in Amazonas (7.0%) and Tocantins (5.8%), in the North [44–46].

In a similar molecular epidemiological study conducted between 2008 and 2009 in which MSM individuals were recruited using the RDS method in the same studied Brazilian cities [47], higher subtype B (81.8%), as well as smaller percentage of subtypes C (7.7%), recombinants (6.9%) and F1 (3.6%) were verified when compared with the present study. Geographically, in the MSM study high subtype B predominance were detected in all regions (90.6% in the northeast, 89.5% in the central west and 80.0% in the southeast), except for the south where it constituted 50% [47]. Herein the dominance of subtype B was verified only in the southeast (70%). In central west and northeast it represents about 50% of the HIV-1 infections, showing that the prevalence of subtypes may differ according to the key study population in Brazil. These results reinforce previous findings of higher prevalence of sub-subtype F1 and BF1 recombinants when compared to other key populations at higher risk [17,18]. Nevertheless, in Brazil multiple unique BF recombinant forms play a role in dynamic epidemic, and only CRF28_29_BF has higher prevalence in São Paulo. Notwithstanding, but they did not exhibit a strong founder effect and have showed a decreasing prevalence over time [48].

In the neighboring countries, HIV-1 molecular epidemic is manly driven by subtype B and BF recombinant forms. In Argentina and Uruguay, most recombinant forms were CRF12 and their related forms, as well as CRF17 and CRF38 also have importance in the epidemic in Argentina and in Uruguay, respectively [49,50]. In Argentina the recombinant forms were spread among the infected individuals independently of the transmission route. However CRF12-related strains were detected in 100% of the IDUs in a study carried out by Espinosa et al. 2004, who associated this group to the spread of BF recombinants to the heterosexual population [51].

Interestingly, some clustering of RDS groups and reference sequences searched by BLAST were detected in *env* and *pol* analysis. In the *pol* ML, the samples from group 1 were collected in PE, as well as the two reference sequences from blood donors that clustered with them. These RDS samples probably belong to the CRF70 or CRF71_BF that were recently characterized in this region, and that presents Fpol/Benv pattern of recombination in the analyzed genomic region. These samples also cluster with reference sequences from this CRFs but with aLRT<0.90. In *env* ML, The group 1 cluster with two HSH sequences demonstrating transmission networks between drug users and HSH individuals from Rio de Janeiro. Unfortunately, these sequences were not available for the *pol* region. The groups 2 and 3 had clustered with non-Brazilian samples about which there is no information about transmission categories. The reference sequence that cluster with PE91 and PE97 is from Italy and also cluster with these sequences in pol but with an aLRT<0.90. Is important to point out that none Brazilian sequences were retrieved from the BLAST *env* search. The cluster from BA sequences (3) included a sample from Thailand but only in the *env* region.

A temporal increasing tendency of TDRM acquisition was observed in previous studies with larger number of HIV-1 seropositive individuals, covering several Brazilian cities, varying from 5.4% in 2003 [52] to 8.1% in 2009 [53]. More recently, Soares et al., 2014 [46] detected 5%-15% of TDRM in Manaus and Itajaí and >15% in the other geographic regions under analysis [54]. Herein, moderate level of TDRM was detected (12.2%) in the present study among DU from different Brazilian cities. As drug users usually present low adherence to the treatment and clinical follow-up, we expected to observe higher rates of TDRM in comparison to studies conducted with others HIV-1 vulnerable populations. However, a previous RDS study including drug naïve MSM individuals from different Brazilian cities found 21% of TDRM [47]. Moreover, a study conducted from 2002 to 2007 in European countries revealed that MSM have significantly higher TDRM prevalence when compared to heterosexuals and IDUs [14]. Previous studies conducted by our group in IDUs from Rio de Janeiro at two time periods (1994–1997 and 1999–2001) observed an increase of PI (0 to 7.9%) and NNRTI (4.0% → 11.4%), as well as a decrease of NRTI mutations frequencies (19.2% → 16.0%) [18]. Herein, resorting data of drug users from RJ, including samples collected in 2009, we observed intermediary values of PI (2.9%), NRTI (6.1%) and NNRTI (6.1%) mutations prevalence in comparison to the previous ones.

The study had some limitations, such as insufficient biological material in the dried blood spots, preventing the enrollment of part of the HIV positive individuals in the molecular analysis and the lack of information concerning the use of HAART in the questionnaire, which did not integrate socio-demographic and behavioral data with clinical information.

Notwithstanding, the study has important strengths, as the sucessfully employment of the RDS methodology in the recruitment of the DU in a short period of time, making possible to perform virological surveillance of DU in eight cities from distinct Brazilian regions and to detect clusters in the phylogenetic analyses that in some contexts were found to be coincident with the networks made evident by the RDS chain-referral structure.

## Supporting Information

S1 Fig Phylogenetic tree analysis of the 132 HIV-1 positive individuals identified by RDS based on the *pol* (*PR/RT*) region. The phylogenetic inferences were performed by the ML algorithm under GTR+I+Γ4 nucleotide substitution model using PhyML. The scale represents number of substitutions per site. The color of the branches represents the Brazilian States from where the individual’s sequences originated, according to the legend in the figure. aLRT superior than 0.90 were depicted. CRF28/29_BF samples with additional recombination breakpoint were represented as second generation (2nd gen.).
(TIF)

S2 Fig Phylogenetic tree analysis of the 132 HIV-1 positive individuals identified by RDS based on the C2-V3 *env* region. The phylogenetic inferences were performed by the ML algorithm under GTR+I+Γ4 nucleotide substitution model using MEGA v6.0 package. The scale represents number of substitutions per site. The color of the branches represents the Brazilian States from where the individual’s sequences originated, according to the legend in the figure. aLRT superior than 0.90 were depicted.
(TIF)

S3 Fig Schematic drawing showing breakpoint patterns of all HIV-1 recombinant patterns detected in the analyses of the *env- C2-V3* (6922–7277 relative to the HXB2) and *pol-PR/RT* (2265–3519 relative to the HXB2) regions Breakpoint positions were obtained using bootscanning and informative site analysis for intragenic recombinant sequences. The studied regions were colored according to the subtype as shown in the figure legend. The fragments not analyzed are represented in white. Genomic structures were drawn by using the Recombinant Draw Toll available in the Los Alamos homepage (http://www.hiv.lanl.gov/content/hiv-db/DRAW_CRF/recom_mapper.html).
(TIF)
