# Supplementary figures and images for: Assessing the HIV-1 Epidemic in Brazilian Drug Users: A Molecular Epidemiology Approach
Source: PLoS One. 2015 Nov 4;10(11):e0141372. doi: 10.1371/journal.pone.0141372 (PMC4633026; doi:10.1371/journal.pone.0141372)

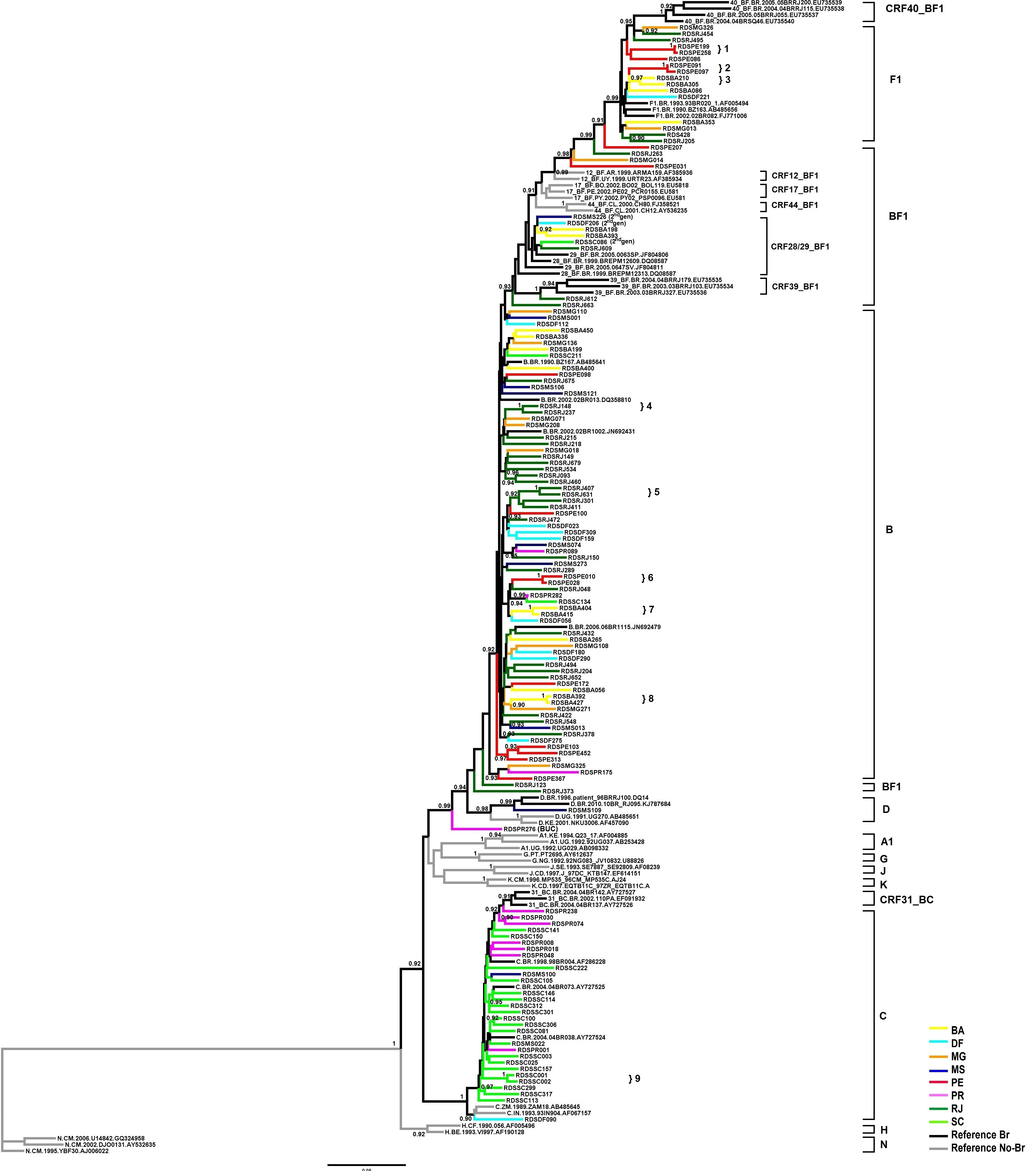

Supplement: S1 Fig — The phylogenetic inferences were performed by the ML algorithm under GTR+I+Γ4 nucleotide substitution model using PhyML. The scale represents number of substitutions per site. The color of the branches represents the Brazilian States from where the individual’s sequences originated, according to the legend in the figure. aLRT superior than 0.90 were depicted. CRF28/29_BF samples with additional recombination breakpoint were represented as second generation (2nd gen.). (TIF) [file pone.0141372.s001.tif]

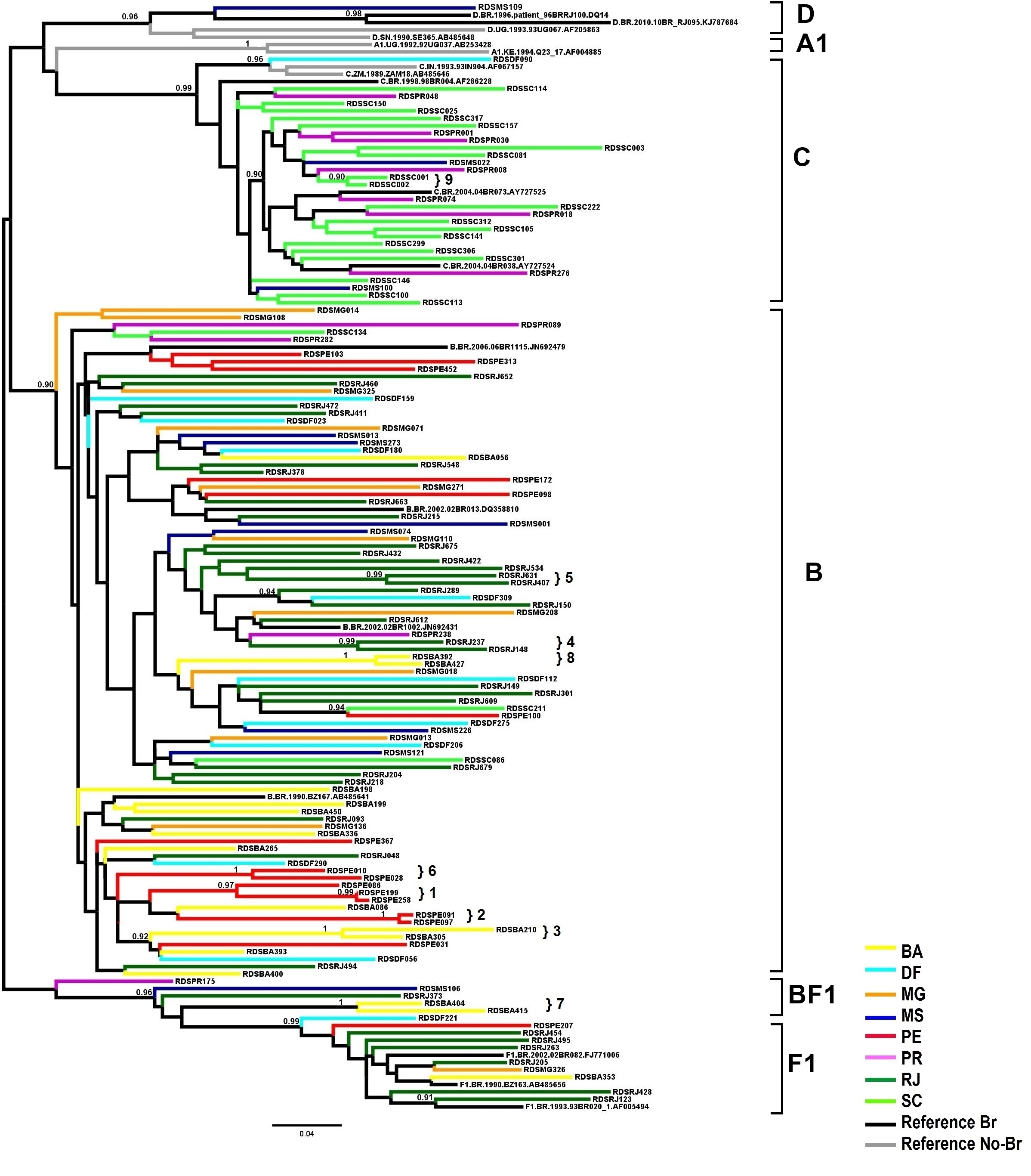

Supplement: S2 Fig — The phylogenetic inferences were performed by the ML algorithm under GTR+I+Γ4 nucleotide substitution model using MEGA v6.0 package. The scale represents number of substitutions per site. The color of the branches represents the Brazilian States from where the individual’s sequences originated, according to the legend in the figure. aLRT superior than 0.90 were depicted. (TIF) [file pone.0141372.s002.tif]

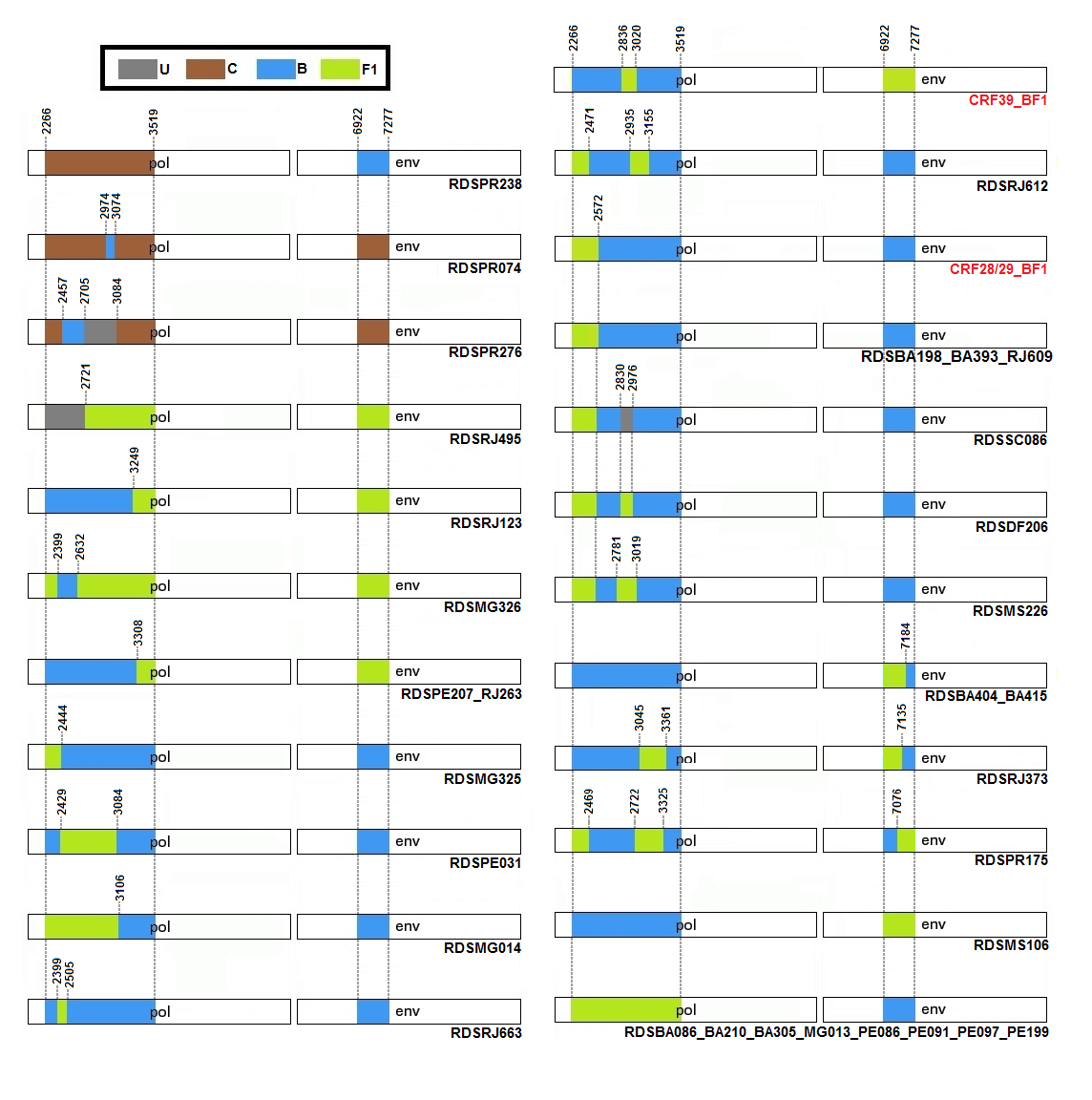

Supplement: S3 Fig — The studied regions were colored according to the subtype as shown in the figure legend. The fragments not analyzed are represented in white. Genomic structures were drawn by using the Recombinant Draw Toll available in the Los Alamos homepage (http://www.hiv.lanl.gov/content/hiv-db/DRAW_CRF/recom_mapper.html). (TIF) [file pone.0141372.s003.tif]
